# *Notes from the Field:* Acute Hepatitis A Virus Infection Among Previously Vaccinated Persons with HIV Infection — Tennessee, 2018

**DOI:** 10.15585/mmwr.mm6814a3

**Published:** 2019-04-12

**Authors:** Julia Brennan, Kelly Moore, Lindsey Sizemore, Samantha A. Mathieson, Carolyn Wester, John R. Dunn, William Schaffner, Timothy F. Jones

**Affiliations:** ^1^Epidemic Intelligence Service, CDC; ^2^Communicable and Environmental Diseases and Emergency Preparedness, Tennessee Department of Health; ^3^Department of Health Policy, Vanderbilt University Medical Center, Nashville, Tennessee.

Complete immunization against hepatitis A requires 2 doses of a monovalent vaccine or 3 doses of a combined hepatitis A and hepatitis B vaccine; approximately 90% of vaccinated persons achieve protective antibody levels after a single dose of either product (*1*). However, persons living with human immunodeficiency virus (HIV) infection might not develop the same level of immunity after hepatitis A virus (HAV) vaccination as do immunocompetent persons (*2*,*3*). Compared with immunocompetent persons, seroconversion rates among persons with HIV infection are lower and are further affected by CD4 count and HIV viral load at the time of the first dose of vaccine (*3*). In addition, time to seroconversion is longer (*3*), and duration of protection wanes earlier (*4*) among persons with HIV infection. During an outbreak, evaluating predictors of a better vaccine response (CD4 count and HIV viral load at the time of first vaccination) is generally not feasible. Routine assessment of immune response after vaccination is not recommended for persons in general, nor for those with HIV infection (*1*); therefore, providers use a documented history of HAV vaccination to guide decisions regarding administration of HAV postexposure prophylaxis (PEP). However, compared with vaccination among the general population, a previous hepatitis A vaccination in persons with HIV infection after a high-risk exposure (e.g., household member or sexual contact) might not reliably protect against illness. The Tennessee Department of Health (TDH) sought to determine the frequency at which persons with HIV infection who were previously vaccinated for hepatitis A developed HAV infection during an HAV outbreak.

Confirmed HAV cases reported to TDH during an ongoing HAV outbreak during December 1, 2017–September 20, 2018, were reviewed to identify patients with HIV coinfection. Data gathered from case report forms, surveillance databases, and medical records were used to evaluate HIV status and HAV vaccination history.

Among 249 confirmed cases of HAV infection, 11 (4%) occurred among persons with HIV infection, six of whom had received a partial or complete vaccination series before acute HAV infection (Table). All six patients were men. Among three patients who had received a monovalent vaccine, one (patient A) completed a 2-dose series 3 years before HIV diagnosis and 7 years before acute HAV infection. A second patient (patient B) received both doses 5 years before the onset of acute HAV infection. A third patient (patient C), who had received 1 dose 44 days before being identified as a sexual contact of a person with acute HAV infection, received PEP consisting of 1 dose of monovalent vaccine at 7 days and immune globulin (IG) at 14 days after the latest possible exposure but developed illness 6 days after PEP was completed. All three patients who received combined hepatitis A and hepatitis B vaccine (patients D, E, and F) had received only 1 or 2 doses of the 3-dose series. Five of six patients initiated vaccination after HIV diagnosis, although all six patients had an indication for routine HAV vaccination that predated HIV diagnoses, including identifying as a man who had sex with men or use of recreational drugs (*1*).

**TABLE:** Characteristics of six persons living with human immunodeficiency virus (HIV) infection and acute hepatitis A virus (HAV) infection who had received partial or complete hepatitis A vaccination — Tennessee, December 1, 2017–September 20, 2018

Characteristic	Patient					
	A	B	C	D	E	F
Age (yrs)	29	31	30	38	36	55
Interval from HIV diagnosis to HAV infection	5 yrs	9 yrs	3 mos	4 mos	3 mos	5 yrs
No. of doses monovalent HAV vaccine received	2/2	2/2	1/2	—	—	—
No. of doses combined HAV and hepatitis B vaccine received	—	—	—	2/3	1/3	2/3
HAV vaccination status	Full	Full	Partial	Partial	Partial	Partial
Received postexposure prophylaxis	No	No	Yes	No	No	No
Interval from first HAV vaccine dose to HAV infection	8 yrs	8 yrs	2 mos	2 mos	1 mo	5 mos
Interval from most recent HAV vaccine dose to HAV infection	7 yrs	5 yrs	13 days	6 days	1 mo	3 mos
CD4 count before first HAV vaccine dose*	Vaccinated 3 yrs before HIV diagnosis	358	887	532	862	342
HIV viral load before first HAV vaccine dose^†^	Vaccinated before HIV diagnosis	1,886	154	136	2,554	20
CD4 before HAV infection	N/A	243	779	403	N/A	225
HIV viral load before HAV infection	N/A	28,474	20	26	N/A	20

**Abbreviation:** N/A = not available.

* CD4 count >500 cell/mm^3^ indicates healthy immune function.

^†^ HIV viral load <50 copies/mL indicates viral suppression.

Previous vaccination for hepatitis did not reliably provide protection among some persons with HIV infection. Approximately half of the patients with HAV and HIV infections were previously vaccinated. The Advisory Committee on Immunization Practices does not currently address specific PEP considerations for persons with HIV infection who have been fully vaccinated against hepatitis A (*1*). CDC guidelines recommend IG and a dose of vaccine as PEP for hepatitis A for previously unvaccinated persons who are immunocompromised, including persons with HIV infection (*2*). These findings support the consideration by providers to administer IG as PEP for all persons with HIV infection who experience high-risk exposure to a person with HAV infection, regardless of the exposed persons prior vaccination history or immune status.
